# Postural Control While Walking Interferes With Spatial Learning in Older Adults Navigating in a Real Environment

**DOI:** 10.3389/fnagi.2020.588653

**Published:** 2020-11-12

**Authors:** Catherine Persephone Agathos, Stephen Ramanoël, Marcia Bécu, Delphine Bernardin, Christophe Habas, Angelo Arleo

**Affiliations:** ^1^Sorbonne Université, INSERM, CNRS, Institut de la Vision, Paris, France; ^2^University of Côte d’Azur, LAMHESS, Nice, France; ^3^Vision Sciences Department, Essilor International R&D, Paris, France; ^4^Essilor Canada Ltd., Montreal, QC, Canada; ^5^CHNO des Quinze-Vingts, INSERM-DGOS CIC 1423, Paris, France

**Keywords:** aging, cognitive-motor interference, spatial learning, navigation, walking speed, postural control, brain atrophy, parietal cortex

## Abstract

Cognitive demands for postural control increase with aging and cognitive-motor interference (CMI) exists for a number of walking situations, especially with visuo-spatial cognitive tasks. Such interference also influences spatial learning abilities among older adults; however, this is rarely considered in research on aging in spatial navigation. We posited that visually and physically exploring an unknown environment may be subject to CMI for older adults. We investigated potential indicators of postural control interfering with spatial learning. Given known associations between age-related alterations in gait and brain structure, we also examined potential neuroanatomical correlates of this interference. Fourteen young and 14 older adults had to find an invisible goal in an unfamiliar, real, ecological environment. We measured walking speed, trajectory efficiency (direct route over taken route) and goal fixations (proportion of visual fixations toward the goal area). We calculated the change in walking speed between the first and last trials and adaptation indices for all three variables to quantify their modulation across learning trials. All participants were screened with a battery of visuo-cognitive tests. Eighteen of our participants (10 young, 8 older) also underwent a magnetic resonance imaging (MRI) examination. Older adults reduced their walking speed considerably on the first, compared to the last trial. The adaptation index of walking speed correlated positively with those of trajectory efficiency and goal fixations, indicating a reduction in resource sharing between walking and encoding the environment. The change in walking speed correlated negatively with gray matter volume in superior parietal and occipital regions and the precuneus. We interpret older adults’ change in walking speed as indicative of CMI, similar to dual task costs. This is supported by the correlations between the adaptation indices and between the change in walking speed and gray matter volume in brain regions that are important for navigation, given that they are involved in visual attention, sensory integration and encoding of space. These findings under ecological conditions in a natural spatial learning task question what constitutes dual tasking in older adults and they can lead future research to reconsider the actual cognitive burden of postural control in aging navigation research.

## Introduction

Spatial navigation is a daily activity, essential for independence, safety and quality of life. It is a complex task, relying on the creation and updating of internal representations of one’s environment and spatial relationships within it, including the self (Wolbers and Hegarty, 2010). Spatial learning is an attentionally demanding task (Lindberg and Gärling, 1982; Albert et al., 1999; Rand et al., 2015). Postural control, including walking, also requires attentional resources, especially for older adults (Holtzer et al., 2006; Yogev-Seligmann et al., 2008). Yet research on navigation in older adults rarely considers the potential interference between controlling one’s body and holding a mental representation of the environment and of their own state within it. With the current paper, we argue that the deterioration of navigational abilities observed in older adults (Moffat, 2009; Klencklen et al., 2012; Lester et al., 2017) might not be accounted solely by perceptual and cognitive age-related deficits, but also by how these changes interfere with deficits in postural control (at the motor and cognitive level).

Successful spatial navigation requires manifold perceptual and cognitive faculties (e.g., in the exploitation of optic flow and processing of sensory cues, working memory, attention allocation and mental manipulation of spatial information). This activity is therefore severely affected by aging, given the deteriorations in these faculties (Moffat, 2009). Navigation abilities are indeed increasingly being studied as indicators in the development of pathologies such as Alzheimer’s disease (Gazova et al., 2012). We also know that exploiting idiothetic cues (i.e., efferent motor, and reafferent proprioceptive and vestibular information) during active exploration, improves spatial learning and navigation performance (Waller et al., 2004; Chrastil and Warren, 2012). Older adults benefit from such self-motion cues, compared to situations where this information is lacking (Mahmood et al., 2009; Adamo et al., 2012; Harris and Wolbers, 2012 but see also Taillade et al., 2016). However, this population also seems to neglect idiothetic cues to a certain extent, due to the noisiness of the signals following age-related degradation (Shaffer and Harrison, 2007; Anson and Jeka, 2016; Kamil et al., 2018), and due to the concurrent reliance on visual information (Agathos et al., 2015, 2017; Alberts et al., 2019). In addition, the spatial and motor representation of older adults’ body is altered by the aging process, rendering their body schema and subjective perception of motor abilities inconsistent with respect to their actual state (Personnier et al., 2010; Boisgontier and Nougier, 2013; Lafargue et al., 2013). Finally, static (standing) and dynamic (locomotion) postural control involves a cognitive, attentionally demanding component (Woollacott and Shumway-Cook, 2002). This component increases with age (Amboni et al., 2013), and even “simple” walking constitutes a more cognitively demanding task for older adults (e.g., Holtzer et al., 2011; Zwergal et al., 2012).

Cognitive-motor interference (CMI) is the phenomenon whereby concurrent cognitive and motor tasks require shared or synchronous attentional resources or information-processing neural pathways. CMI is particularly sensitive to aging (Lindenberger et al., 2000; Yogev-Seligmann et al., 2008; Beurskens and Bock, 2012; Montero-Odasso et al., 2012), given the multiple cognitive and sensorimotor alterations that occur in older age (Seidler et al., 2010; Paraskevoudi et al., 2018). This is usually studied via the dual task paradigm, which examines changes in participants’ performance in separately (single) versus simultaneously (dual) executed tasks, or by comparing dual task performance between different levels of difficulty in one of the two tasks (Woollacott and Shumway-Cook, 2002). The resulting CMI depends on both the nature of each task and the population studied. For example, studies showed that visual/spatial cognitive tasks interfere with postural control to a greater extent than other cognitive tasks (Kerr et al., 1985), especially among older adults (Maylor and Wing, 1996; Bock, 2008; Menant et al., 2014).

Very few studies examined CMI in the context of navigation, by investigating the cognitive demands of motor tasks interacting with spatial learning and wayfinding (typically varying the difficulty of the cognitive or motor component of the activity). An early study by Kerr et al. (1985) suggested that standing postural control and spatial memory may share cognitive resources, as demonstrated by a reduction in performance on the Brooks spatial memory task while standing compared to sitting in healthy young adults. In another study, younger and older men performed a wayfinding task in virtual maze-like museums projected on a screen and coupled to a treadmill (Lövdén et al., 2005). The participants performed the task while walking with or without postural support (a handrail placed in front of them). Older adults’ navigational place learning performance improved when the postural demands of walking were alleviated with the aid of the handrail, reducing the observed age-related differences without the postural support. The same group later explored the effect of cognitive demand of different navigation aids for older adults on their navigation performance and walking variability (Schellenbach et al., 2010). Faced with maze-like zoos this time, while walking on a treadmill, younger and older men performed a wayfinding task without any assistance, with a virtual guide (a red line on the path they should follow) or with an overview map appearing in the corner of the display. While both aids improved older adults’ navigation performance, this amelioration was more important with the virtual guide compared to the presence of the map, which failed to eliminate attentional resource competition for these participants. Finally, Rand et al. (2015) explored the attentional demands of spatial learning of young adults in a real indoor environment in a series of experiments. These authors’ premise was that low vision calls for additional attentional demands for safe postural control (what they termed mobility monitoring), which can interfere with spatial memory performance during navigation. They showed that being physically guided by an experimenter while walking with simulated low vision (wearing blur goggles) reduced attentional resource demands as revealed by improved spatial memory. This finding was replicated by the authors in an older adult population as well (Barhorst-Cates et al., 2017). Altogether, these studies support the hypothesis that postural control and spatial navigation share attentional resources, and therefore CMI in older adults should be considered and better characterized when studying older individuals’ navigation difficulties.

Beyond behavioral observations, neuroimaging findings confirmed the cognitive contributions to walking and postural control (Zwergal et al., 2012; Hamacher et al., 2015). Moreover, age-related reductions in walking speed and increases in gait variability are linked to structural and functional changes in the brain (Rosano et al., 2008, 2010; Rosso et al., 2013; Callisaya et al., 2014; Holtzer et al., 2014; Ezzati et al., 2015). While functional neural correlates of CMI are increasingly being explored (Leone et al., 2017), structural analyses to uncover the neural bases of CMI while walking are extremely limited. A few studies explored dual task walking (e.g., walking while reciting alternate letters of the alphabet or counting backward) in older adults and they found associations with gray matter atrophy across a distributed brain network involving prefrontal, frontal, parietal, occipital, cingulate and thalamic regions (Allali et al., 2019; Tripathi et al., 2019; Wagshul et al., 2019). Thus, there is some overlap between areas impacted by age that are involved in postural control (including while dual tasking) and spatial learning and navigation, such as prefrontal and parietal regions (Iaria et al., 2003; Moffat et al., 2007; Antonova et al., 2009; Moffat, 2009; Piefke et al., 2012).

In this study, we analyzed data from an experiment that challenged the premise that older adults have difficulties in employing allocentric navigation strategies (Bécu et al., 2020). We focused on the learning phase of our previous study in order to identify potential indicators of CMI in older adults while exploring and learning an unknown environment. We hypothesized that older adults would show greater changes in their walking speed, compared to young adults, which could be due to cognitive resource sharing between encoding the new environment and postural control. We further explored whether modulations in walking speed while learning the new environment would also be associated with indices of spatial learning. Finally, based on the evidence of age-related brain changes and their association with gait control, we also analyzed MRI data of a sub-sample of participants to uncover possible associations between modulations of walking speed and structural brain changes.

## Materials and Methods

### Navigation Study

#### Participants

Data from 28 participants, 14 young (*M* = 27.3 years, *SD* = 5.4 years, range: 21–37 years, 8 female) and 14 older adults (*M* = 71.4 years, *SD* = 2.8 years, range: 68–77 years, 9 female), were analyzed in this study. The participants were volunteers, part of the SilverSight cohort population at the Vision Institute – Quinze-Vingts National Ophthalmology Centre, in Paris. All screening and experimental procedures were in accordance with the tenets of the Declaration of Helsinki and approved by the Ethical Committee “CPP Ile de France V” (ID_RCB 2015-A01094-45, n. CPP: 16122 MSB). All volunteers provided informed, written consent to participate. Inclusion criteria were: (i) corrected visual acuity of at least 0.15 logMAR (7/10) for young adults, or 0.3 logMAR (5/10) for older adults; (ii) a Mini-Mental State Examination score of 24 or higher; (iii) ability to ambulate without assistance, and (iv) no ophthalmological, otological/vestibular or neurological disorders as assessed with relevant screenings. Descriptive demographic information of each age group is presented in Table 1, including education level, height and weight. A subset of visuo-cognitive screening assessments carried out for the original navigation study (Bécu et al., 2020) was used here to examine potential links with navigation performance: the Figural memory test (long-term figural memory), Corsi block tapping test (working memory span), Perspective taking/Spatial orientation test (visuospatial ability), the Trail making test (part A – processing speed), 3D mental rotation test and the State-trait anxiety inventory (state scale). Age group scores on these tests in addition to visual acuity and MMSE score are provided in Table 2.

**TABLE 1:** Descriptive demographic information of participants in each age group.

	**Young adults**	**Older adults**
Age – years	27.3 ± 5.4	71.4 ± 2.8
Height – meters	1.70 ± 0.09	1.65 ± 0.07
Weight – kilograms	64.3 ± 11.9	68.2 ± 10.8
Education level according to the International Standard Classification of Education - ISCED 2011 (UNESCO Institute for Statistics, 2013)	Level 4 or above: 14/14	Level 2: 2/14 Level 3: 2/14 Level 4 or above: 10/14

*Mean values with standard deviations are presented except for the level of education. For reference, Level 2 corresponds to a lower secondary education diploma or equivalent; Level 3 corresponds to a high school (upper secondary level) diploma or equivalent; Level 4 corresponds to a post-secondary non-tertiary education diploma.*

**TABLE 2:** Mean scores with standard deviations on the visuo-cognitive screening tests for each age group.

	**Young adults**	**Older adults**
Mini Mental State Examination - MMSE Score	2*29.7 ± 0.5	2*28.4 ± 1.7*
Visual acuity – logMAR	−0.250.04	−0.100.06*
Processing speed – seconds (Trail Making Test – Part A)	2*13.97 ± 1.64	2*25.88 ± 4.77*
Spatial working memory span – number of blocks (Corsi Block Tapping Test)	2*6.09 ± 1.38	2*4.21 ± 0.89*
Perspective taking – mean angular deviation in degrees	2*20.29 ± 9.54	2*49.15 ± 20.55*
3D mental rotation – number of correct items	2*19.36 ± 2.84	2*9.29 ± 3.52*
State-trait anxiety inventory – State scale score	2*28.45 ± 5.57	2*30.07 ± 7.05

*p < 0.05.

#### Experimental Setup

The experiment was performed in a real environment using the Streetlab platform at the Vision Institute in Paris^1^. The environment consisted of an 8.55 m by 4.30 m laboratory with 19 panels covering the walls, providing real-world textures (3 m high, of variable width) imitating a street-like environment such as brick walls, doors and windows. The floor of the room was covered with a black linoleum surface and it was free of any obstacles. A homogeneous illumination (195 lux) and street-like multisource sounds were provided during the whole experiment to increase immersion. A complete account of experimental setup, procedure and data acquisition details has been described previously (Bécu et al., 2020).

#### Data Acquisition

An optoelectronic motion capture system (10 infra-red cameras, model T160, VICON Motion Systems Inc., Oxford, United Kingdom) was used to record participants’ kinematics at a sampling frequency of 120 Hz. The cameras were positioned above the panels and placed symmetrically to avoid spatial cueing. During the experiment, participants were equipped with a tight black suit, onto which 39 retro-reflective markers were placed following the Vicon Plug-In Gait model. Movement of the right eye was recorded by a video-based eye-tracker (Mocaplab, Paris) at 60 Hz. The eye-tracking camera was mounted on a light frame that permitted the participants to wear their own glasses, if necessary, during the experiment. Participants habitually wearing far-vision lenses were encouraged to keep their glasses on during the experiment.

A calibration to obtain the 3D gaze vector in the reference frame of the environment involved computing the center of rotation of the eye, relative to four retro-reflective markers positioned on the eye-tracker frame, and its correspondence with the laboratory space coordinates. Correspondence between the eye position in the eye-tracker camera coordinates and the laboratory coordinates was calculated using an ellipsoidal calibration grid (145 cm × 100 cm) composed of 25 markers, placed at eye level, approximately one meter from the participant. Additionally, in order to correct for potential drift occurring over the course of the experiment, a two-point drift correction was performed before each trial.

### Procedure

**FIGURE 1:**
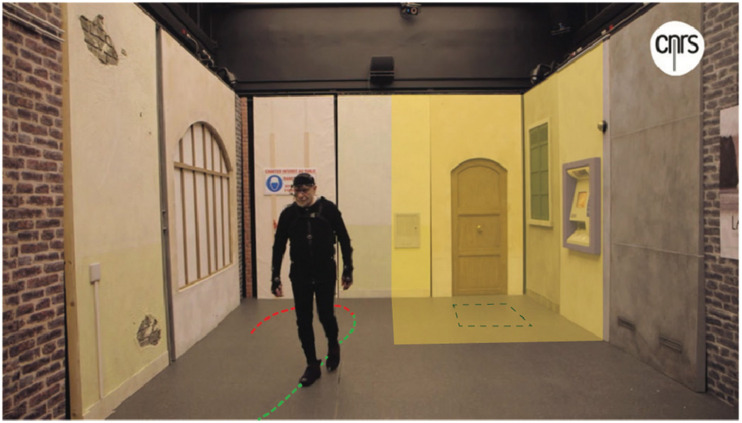
The real, ecological experimental setup. In the Streetlab platform (Vision Institute), a street-like, open-field maze was set up for young and older participants to navigate in a real, yet fully controlled, environment [see section “Materials and Methods” and in Bécu et al. (2020)]. Whole-body and eye movements were recorded as participants navigated through the obstacle-free environment. The rectangle in the highlighted area indicates the unmarked goal location (80 cm × 80 cm). The highlighted surfaces indicate the region in which fixations were defined as within the goal area. The dotted line on the floor is an illustration of a trajectory where sequences defined as straight-ahead walking are highlighted in green. Figure adapted from Bécu et al. (2020).

Before their first exposure to the environment, participants were instructed that they would have to navigate as quickly as possible, without running, to an invisible goal location within the experimental environment. At the start of the experiment, the participants were naïve regarding the environmental layout and the goal location. The square goal zone (80 cm × 80 cm) was located in the northwest quadrant of the room during the whole experiment (see Figure 1). All participants were disoriented before each trial. Briefly, the participants sat on a swivel chair with their eyes closed while an experimenter slowly rotated and translated the chair around the room. This movement always started from the center of the room and ended in one of four positions, facing one of three orientations in a pseudo-random order. Participants were then asked to point toward the center of the room (their eyes still closed) for the experimenter to verify that they were truly disoriented [see Supplementary Figure 11 in Bécu et al. (2020)]. All experimenters then left the room and participants were instructed to open their eyes and search for the goal. As soon as the participants’ trajectory crossed the border of the goal zone from any direction, an audio signal was automatically triggered to notify them that the goal was reached. Participants were then instructed to stop moving and close their eyes. Eight learning trials were repeated; a ninth was added for two older participants.

### Data Processing

Eye-tracking data were resampled to 120 Hz and all motion capture data were filtered with a second order, low pass Butterworth filter, with a cut-off frequency at 10 Hz. Participants’ steps were detected for each trial, as well as standing periods. Steps were detected according to heel strikes, identified for each foot by evaluating when the velocity signal of the heel in the vertical axis changed sign. Automatic detection of the above events was manually validated, or corrected when necessary, for each trial. The period between eye opening and the initial step was termed the orientation phase, whereas the period onward was termed the navigation phase of the trial.

Given that participants were free to move as they wished through the environment, we further classified the detected steps according to the curvature of their trajectory and focused our analysis on sequences of straight-ahead walking. Steps were classified as straight-ahead if the difference in heading orientation between the beginning and the end of that step was below 45° in the horizontal plane (yaw rotation). Heading orientation was defined according to the velocity vector of the centroid of the four eye-tracker markers. The straight-ahead walking sequences corresponded to 83.2 ± 5.5% of the navigation phase on average (80.2 ± 5.4% for older adults and 86.3 ± 5.8% for young adults).

Oculomotor data were obtained by computing the gaze vector and its intersection within a 3D model of the environment. We detected gaze fixations by calculating the gaze dispersion vector as the Euclidean distance, in the environment reference frame, between two successive data points. A continuous period of time during which gaze dispersion was below twice the inter-quartile range from the median of the distribution was considered as a candidate fixation. Fixations shorter than 100 ms in duration were excluded from the analysis. To describe how the participants explored the environment, we distinguished fixations directed toward the goal area versus fixations directed elsewhere. The goal area in this analysis constitutes the surfaces of the panels and ground around the goal zone (see highlighted area in Figure 1).

#### Data Analysis

##### Walking speed

Walking speed was calculated for each step on the identified straight-ahead walking sequences. The median was taken over all steps included in straight-ahead walking sequences, for each trial. To avoid biasing toward lower walking speeds by including gait initiation and termination, the first and last steps a participant performed were ignored if they had been included in the straight-ahead walking sequences.

##### Trajectory efficiency

Trajectory efficiency was defined as the ratio of the shortest distance from the participants’ starting position to the center of the goal zone over the total trajectory length of the participant. It is an indicator of whether participants have learnt the environmental layout, with a value closer to 1 (i.e., 100% efficiency) signifying the adoption of a more direct path to the goal.

##### Goal fixations

We calculated the proportion of fixations directed toward the goal area (see Figure 1) with respect to the total number of fixations. Given the variability in trial duration and overall fixation counts, we deemed it more appropriate to examine the proportion of fixations in the goal area rather than the absolute total. A higher proportion of fixations toward the goal area can indicate a more efficient sampling of the environment to locate the goal while learning.

##### Dependent variables

We chose two ways to assess our hypothesis of CMI while learning a novel environment. First, inspired by dual task studies, we examined the change in walking speed between the first and last trial of the experiment, assuming that the cognitive load of encoding the novel environment would diminish over the learning trials. Second, we sought whether postural control (expressed via walking speed) would interact with spatial learning performance (expressed via the trajectory efficiency and goal fixations). We therefore obtained measures of modulation during learning for these three variables (*adaptation indices*, see below) in order to examine whether a correlation existed between them. This would indicate if the trial-to-trial modulation in postural control shares any variance with that of spatial learning performance.

###### Change in walking speed

The percent change in walking speed was calculated, as classically done in dual task studies (Lindenberger et al., 2000). While our protocol was not a dual task study in the conventional sense, our assumption of cognitive load decreasing while learning allowed us to compare two walking situations where one is more cognitively demanding than the other. We examined the change in walking speed (*ws*) between the first and the last trial: $100\times \frac{w\,s_{l\,a\,s\,t}-w\,s_{f\,i\,r\,s\,t}}{w\,s_{l\,a\,s\,t}}$, since, in our context, we considered that the first trial may constitute a situation where the cognitive load of encoding an unfamiliar environment may interfere with postural control for older adults. It corresponded to the first time participants were exposed to the environment, which they had to visually and physically explore in order to find the position of the invisible goal, and remember it for subsequent trials. On the last trial, however, the layout of the environment had been learnt and therefore navigating within it constituted a simpler cognitive task. The last trial could thus be used as a reference for walking speed. In the event that not enough steps were taken during the last trial, we took the penultimate trial to calculate the reference walking speed, provided the learning performance criterion was met (see below). This was the case for nine participants where only one step (excluding gait initiation and termination) was identified as straight-ahead on their final trial. This was because their starting position on the last trial was the closest to the goal.

###### Adaptation indices

**FIGURE 2:**
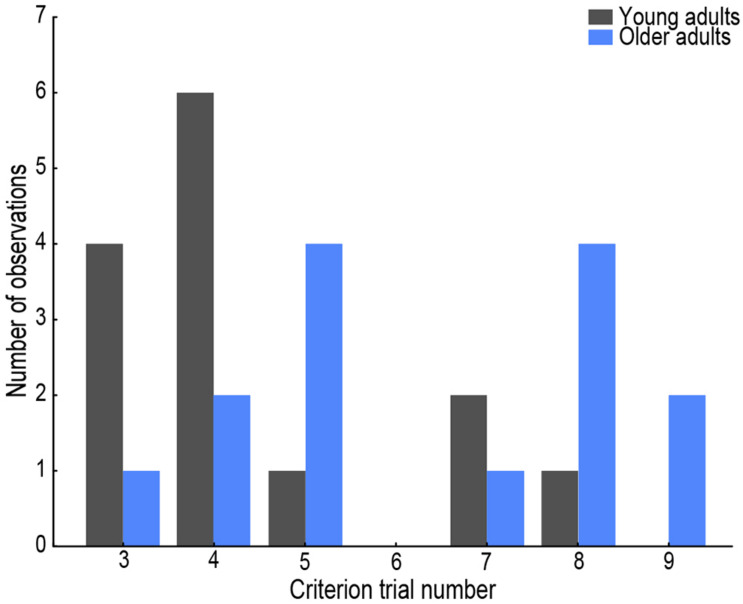
Histogram of trial number that served as the criterion trial per age group. Young adults’ data are presented in gray, older adults’ in blue.

If visual exploration and spatial encoding competed with postural control in terms of cognitive resources, we should observe a change in navigation performance as a function of walking speed during learning. We thus sought to examine whether the adaptation (trial-to-trial change) observed in spatial learning performance, as assessed by the trajectory efficiency and goal fixations, would be associated with the adaptation in walking speed. For this, we first established the trial after which learning was considered to have been achieved. This *criterion trial* was defined as the first trial in which the trajectory efficiency exceeded 0.85 (where 1 indicated taking the most direct route from starting point to the middle of the goal area), provided this measure was above 0.85 on the following trial as well – see Figure 2 for a distribution of the criterion trial number per age group. Then, an adaptation index was calculated for walking speed, trajectory efficiency and goal fixations. This index was a modified version of the spatial learning index commonly described in rodent navigation studies (Tomás Pereira and Burwell, 2015) in order to consider only the trials from the start to the criterion trial in our study. The adaptation index was similar to the slope of a learning curve in that it was an indicator of the “speed” of change in behavior. It was defined as: $\sum_{i=1}^{n}\left(M_{\text{i}}\times S_{\text{i}}\right)$, where *M* is a multiplier, *S* is the variable value on the observed trial and *n* is the criterion trial. The multiplier was calculated taking the mean of the young participants’ score per trial as a control (C), such that *M*_1_ = *C*_1_/*C*_1_, *M*_2_ = *C*_1_/*C*_2_, *M*_3_ = *C*_1_/*C*_3_ etc. As advised for protocols like ours where participants are naïve on the first trial, the first multiplier was then set to zero, *M*_1_ = 0 (Tomás Pereira and Burwell, 2015). Adaptation index interpretation followed the direction of the variable it was calculated for, such that higher values indicated a greater and/or faster change in behavior.

#### Statistical Analysis

To examine age group differences, we first tested normality (Shapiro-Wilk test) and homogeneity of variance (Levene’s test) of our data and we accordingly performed a Student’s *t*-test or a Mann–Whitney *U*-test. These tests were performed on the mean of our three basic behavior variables: walking speed, trajectory efficiency and goal fixations, for descriptive purposes; as well as on our dependent variables: the change in walking speed on the first trial and the adaptation indices of walking speed, goal fixations and trajectory efficiency. Correlations were used to test the possible association between the adaptation index of spatial learning performance measures (goal fixations and trajectory efficiency) and the adaptation index of walking speed. Correlations were also performed on the per cent change in walking speed and the three adaptation indices versus visuo-cognitive test scores. The alpha level for statistical significance was set at *p* < 0.05. Bonferroni corrections were applied to account for multiple testing depending on the number of correlations performed for a given variable – specified in the corresponding “Results” sections below.

### Exploratory Voxel-Based Morphometry Study

#### Participants

Among the 28 healthy participants enrolled in the experiment, 18 also underwent a neuroimaging examination shortly after the navigation study. Exclusions were due to participants either presenting MRI-incompatibility or unavailable for further testing. This sub-sample of participants included 10 young adults (*M* = 27.5 years, *SD* = 5.4 years; range: 21–37 years; 3 male) and 8 older adults (*M* = 70.4 years, *SD* = 2.1 years; range: 68–74 years; 2 male).

#### MRI Acquisition

Magnetic resonance images were acquired using a whole-body 3T Siemens Magnetom Skyra scanner (Siemens Medical Solutions, Erlangen, Germany) with a 64-channel head coil at the Quinze-Vingts National Ophthalmology Hospital in Paris, France. We acquired a T1-weighted high-resolution anatomical volume, by using a three-dimensional magnetization-prepared rapid gradient-echo (MPRAGE) sequence (number of slices = 176, echo time 2.9 ms, repetition time 2300 ms, flip angle = 9°, matrix size = 256 mm × 240 mm × 176 mm, acquisition resolution 1.0 mm × 1.0 mm × 1.2 mm). None of the participants exhibited abnormalities in brain structures.

#### MRI Data Processing

Data processing was performed using SPM12 release 7487 (Wellcome Department of Imaging Neuroscience, London, United Kingdom^2^) implemented in MATLAB 9.4 (Mathworks Inc., Natick, MA, United States). We processed the data via Diffeomorphic Anatomical Registration Through Exponential Lie algebra algorithm (DARTEL) (Ashburner and Friston, 2005; Ashburner, 2007), for the segmentation and normalization steps. The DARTEL segmentation procedure uses a number of tissue probability maps including grey matter (GM), white matter (WM), cerebrospinal fluid (CSF), soft tissue, skull, and non-brain regions of the image. After segmentation, we performed a visual inspection and a quality check of the data, by applying the modules “display one slice for all images” and “check sample homogeneity using covariance” implemented in the VBM12 toolbox^3^. Next, the GM, WM, and CSF tissue classes, obtained during the segmentation step, were used to create a custom template, based on our sample. For each participant, flow fields were computed during template creation to provide the transformation matrix from each native image to the template. Finally, images obtained in the previous step were normalized to the MNI space (voxel size of 1 mm isotropic), modulated and smoothed using an 8-mm full width at half maximum (FWHM) Gaussian kernel. A morphological analysis was performed with the general linear model (Friston et al., 1994) with SPM12 implemented in MATLAB 9.4. We performed a whole brain analysis using the GM images in a multiple regression analysis measuring the relationship between the change in walking speed and GM volume. We used an absolute implicit mask with a threshold value fixed at *p* > 0.2 for GM voxel analyses. To prevent potential bias related to brain size and gender differences in our analysis, TICV (total intracranial volume = GM + WM + CSF) and gender were included in the statistical model, as covariates of no interest. Differences in GM volume were considered significant if they exceeded a voxel-wise threshold of *p* < 0.05 family-wise error (FWE) corrected, with a minimum cluster extent of 10 voxels.

#### Statistical Power Analysis

Given the smaller population sample for the voxel-based morphometry analysis, in addition to the meticulous and conservative analysis described above, we performed a power calculation based on an independent data set involving thirty participants (15 younger and 15 healthy older adults, age range: 30–84 years). This power analysis is described in detail in the Supplementary Material. The results indicate that our sample size of 18 participants was sufficient to detect large effect sizes with enough statistical power (i.e., 80%) but not medium and small effect sizes.

## Results

### Behavioral Results

**FIGURE 3:**
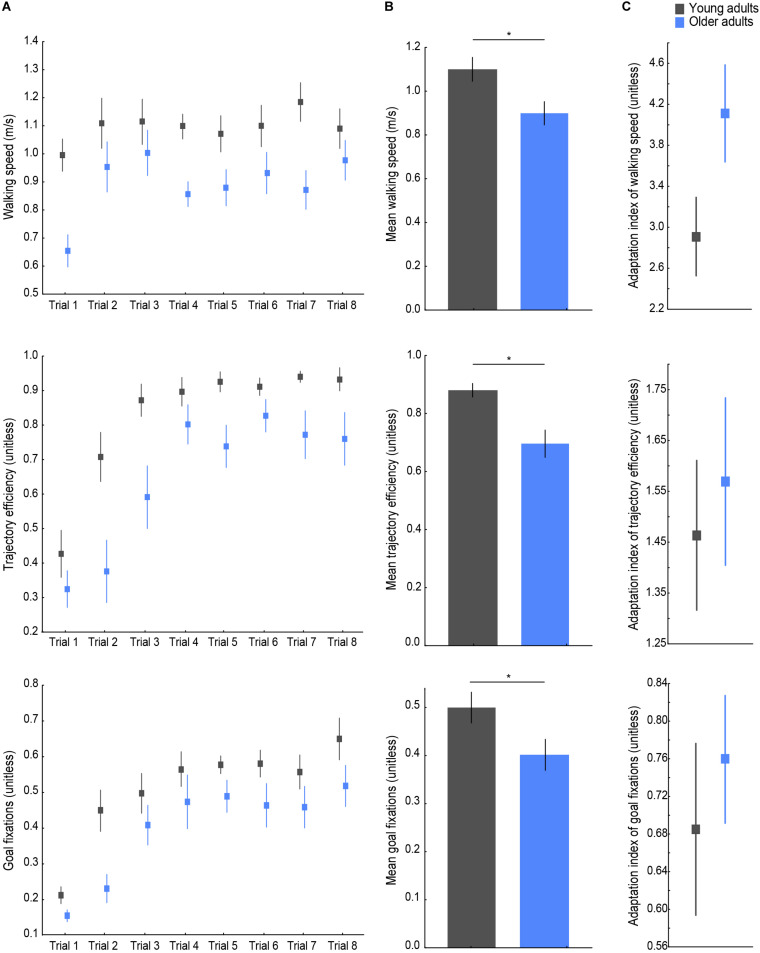
Behavioral navigation variables. **(A)** From top to bottom: walking speed, trajectory efficiency, and goal fixations per age group on each trial. **(B)** Variable means across trials. **(C)** Adaptation indices of each variable. Lines indicate standard errors; asterisks indicate significant differences, *p* < 0.05. Young adults’ data are presented in gray, older adults’ in blue.

Young and older adults’ walking speed, trajectory efficiency, and proportion of goal fixations across all trials are shown in Figure 3A. The walking speed measure was chosen as an indicator of postural control during spatial navigation behavior, the trajectory efficiency to assess goal-directed navigation performance, and the proportion of goal fixations to evaluate the visual sampling strategy during exploration. Age group differences were found on all three average values (see Figure 3B), with a lower mean walking speed [*t*(26) = −2.63, *p* = 0.014, *d* = 0.99], a lower mean trajectory efficiency (*U* = 24, *p* < 0.001, *r* = −0.64), and a lower mean goal fixation proportion [*t*(26) = −2.15, *p* = 0.041, *d* = 0.81] observed in older as compared to young navigators. For the adaptation indices (Figure 3C), a trend was found for the adaptation index of walking speed (*U* = 59, *p* = 0.077, *r* = 0.33), whereby older adults’ larger value indicated a greater/faster change in walking speed across trials. Young and older adults did not differ significantly in terms of the adaptation index of trajectory efficiency (*U* = 91, *p* = 0.77), or the adaptation index of goal fixations [*t*(26) = 0.65, *p* = 0.519] indicating a similar modulation of participants’ trajectory and visual exploration over the trials until the learning criterion was met.

**FIGURE 4:**
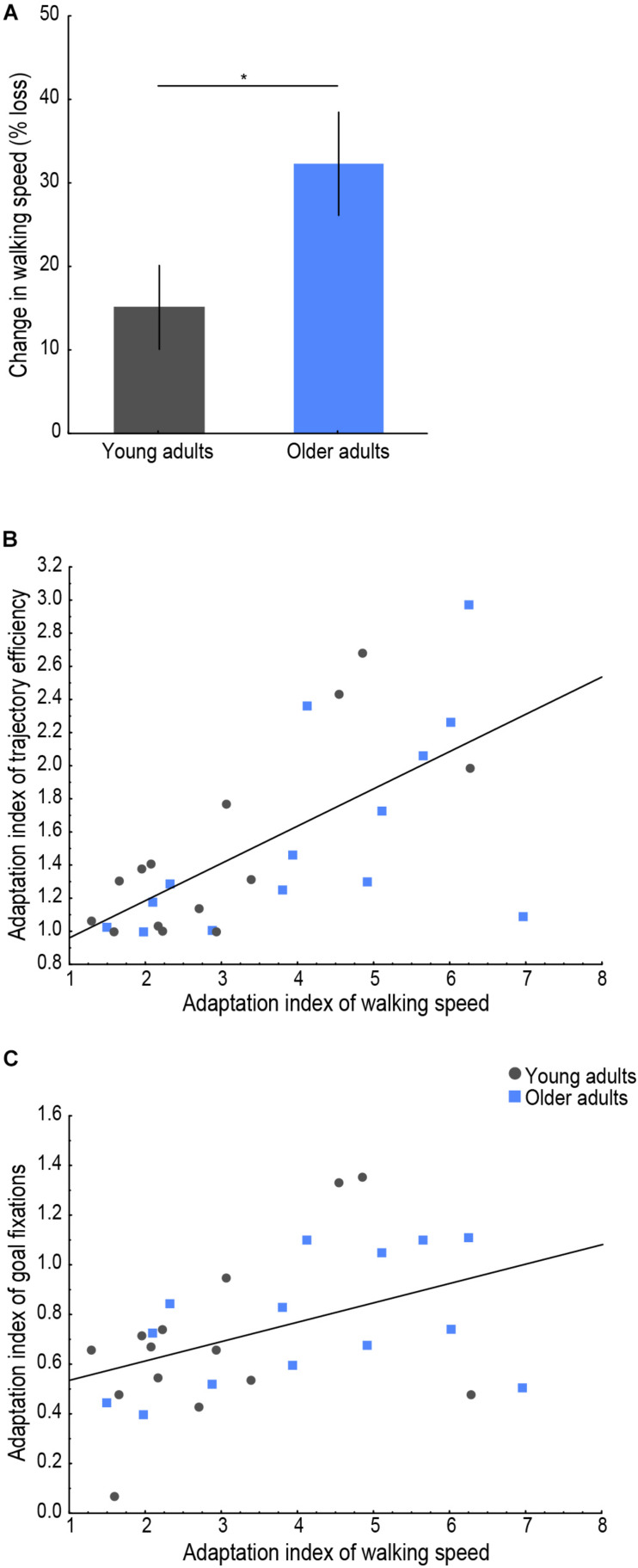
Behavioral indicators of cognitive-motor interference. **(A)** Change in walking speed (% reduction) on the first, compared to the last trial for each age group. Lines indicate standard errors; the asterisk indicates a significant difference, *p* < 0.05. Relationship of the adaptation index of walking speed with **(B)** the adaptation index of trajectory efficiency (*r* = 0.66, *p* < 0.001) and with **(C)** the adaptation index of goal fixations (*r* = 0.44, *p* = 0.019). Young adults’ data are presented in gray, older adults’ in blue.

To examine whether encoding of a new environment while physically exploring it was subject to CMI in older age, we analyzed the percent change in walking speed between the first and last trials and whether a correlation existed between the adaptation index of walking speed and those of trajectory efficiency and goal fixations, as presented in Figure 4. An effect of age was found on the change in walking speed, with older adults showing a larger modulation in their postural control upon first exposure to the environment [Figure 4A; *t*(26) = 2.16, *p* = 0.040, *d* = 0.82], i.e., older adults slowed down to a greater extent on the first trial compared to the last trial compared to young adults. Furthermore, a significant (*p*_*corrected*_ < 0.025) positive correlation was found between the adaptation indices of walking speed and of trajectory efficiency (Figure 4B; *r* = 0.66, *p* < 0.001) as well as between the adaptation indices of walking speed and of goal fixations (Figure 4C; *r* = 0.44, *p* = 0.019). These correlations indicated that learning of the environmental layout during the trials up to the criterion trial was associated with a parallel modulation of the spatial learning performance measures (trajectory efficiency and goal fixations) and postural control (walking speed).

To further explore our interpretation of older adults’ postural adaptation (modulations in walking speed) in order to accomplish the spatial learning task, we also examined potential relationships of the change in walking speed and the adaptation index of walking speed with the visuo-cognitive function tests. All correlation results are presented in Table 3. After correcting for multiple comparisons (*p_*corrected*_* < 0.008), no correlation reached significance. Trends were found, however, between the change in walking speed and figural learning score (Figural Memory Test: *r* = −0.46, *p* = 0.022) and between the adaptation index of walking speed and perspective taking (Perspective taking/Spatial orientation test: *r* = 0.45, *p* = 0.025). These findings indicated a tendency of participants requiring a greater change in walking speed on the first trial (i.e., slow down with respect to the last trial) also scoring lower on the Figural Memory Test and that the trial-to-trial modulation in walking speed was somewhat proportional to participants’ ability to imagine different perspectives during a spatial orientation task.

**TABLE 3:** Correlations between variables expressing the modulation in walking speed and visuo-cognitive screening tests.

	**Change in walking speed between first and last trials**	**Adaptation index of walking speed**
Processing speed (Trail Making Test – Part A)	*r* = 0.40 *p* = 0.047	*r* = 0.20 *p* = 0.335
Long-term figural memory (Figural Memory Test)	*r* = −0.46 *p* = 0.022	*r* = −0.19 *p* = 0.371
Working spatial memory span (Corsi Block Tapping Test)	*r* = −0.32 *p* = 0.125	*r* = −0.27 *p* = 0.185
Perspective taking	*r* = 0.22 *p* = 0.285	*r* = 0.45 *p* = 0.025
3D mental rotation	*r* = −0.22 *p* = 0.288	*r* = −0.31 *p* = 0.135
State-trait anxiety inventory	*r* = 0.18 *p* = 0.394	*r* = 0.27 *p* = 0.197

### Association Between Change in Walking Speed and Cortical Atrophy

**FIGURE 5:**
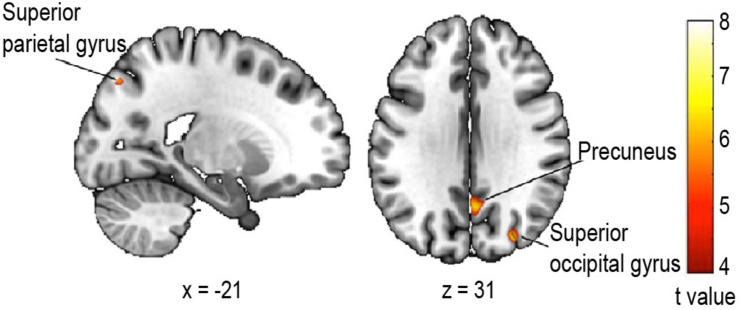
Regression analysis between gray matter volume and change in walking speed. Areas where the negative correlation proved significant: Superior parietal lobule, left hemisphere, *t* = 7.70; peak coordinate *x* = –20, *y* = –76, *z* = 45 (*k* = 10); Precuneus, right hemisphere, *t* = 7.41 peak coordinate *x* = 4, *y* = –60, *z* = 30 (*k* = 52); Superior aspect of occipital lobe on the lateral surface, right hemisphere, *t* = 7.47 peak coordinate *x* = 30, *y* = –81, *z* = 32 (*k* = 28). Statistical threshold *p* < 0.05 FWE (Family Wise Error) corrected for multiple comparisons, extended threshold fixed at *k* = 10 voxels.

Finally, we tested whether the change in walking speed on the first trial compared to the last was associated with gray matter atrophy in older adults, by performing a voxel-based morphometry study. The multiple regression analysis revealed a significantly decreased gray matter volume in parietal and occipital regions (Figure 5) related to an increased change in walking speed. More specifically, we observed a reduced gray matter volume of the left superior parietal lobule [BA 7, *t*(14) = 7.70, *p*_*corrected_FWE*_ = 0.038, *r* = 0.90, *k* = 10], the superior aspect of the occipital lobe on the lateral surface of the right hemisphere [BA 39, *t*(14) = 7.47, *p*_*corrected_FWE*_ = 0.030, *r* = 0.89, *k* = 28] as well as of the right precuneus [BA 31, *t*(14) = 7.31, *p*_*corrected_FWE*_ = 0.022, *r* = 0.89, *k* = 52].

## Discussion

In this paper, we hypothesized that visually and physically exploring a real, unknown environment in order to locate and reach a target position could be prone to cognitive-motor interference (CMI) in older adults. Aging leads to alterations and/or deficits in perception, motor control, and cognition. Therefore, safe and successful navigation for older adults also means larger attentional demands in the execution of ambulation, spatial learning, and reorientation. We found three indicators in support of our hypothesis: (i) a greater change in walking speed between the first and last spatial learning trials for older as compared to young adults, (ii) a positive correlation between the modulation in walking speed and modulations in gaze behavior and walking trajectory over trials while learning (via the adaptation indices), and (iii) a negative correlation between the change in walking speed and gray matter volume in associative areas of the brain. While exploratory, to the best of our knowledge this study is the first to address CMI with healthy older adults in the context of navigation in an ecological setting.

### Interaction of Postural Control With Spatial Learning

Examining the behavior of our participants during the navigation task, we sought to uncover a potential interaction between postural control and spatial learning. We considered that the first trial, upon initial exposure to the unknown environment, would be most likely to provoke CMI for older adults, which is akin to dual tasking: visually exploring and attempting to encode the goal location while also controlling their body through the environment. Walking speed is a common measure of postural control and indicative of gait alterations in dual task walking protocols (Smith et al., 2016). Further, walking speed is often used as a clinical marker of frailty, fall risk and future cognitive decline (Montero-Odasso et al., 2012) and it is also an index sensitive to age-related structural brain changes (Amboni et al., 2013). In our study, the per cent change in walking speed on the first trial with respect to the last was significantly greater for older adults. These participants appeared to need to slow down in order to explore and process the environmental features. Importantly, this finding was despite specifically examining sequences identified as straight-ahead walking. This methodological decision was made to avoid biasing toward slower walking speeds by including turning and stopping behavior within a trial, thus operating against our hypothesis.

Many theoretical models have been suggested to describe CMI (Beurskens and Bock, 2012; Wollesen et al., 2019). On the one hand, the reduction in walking speed that we observed could be considered as a change whereby the task of visual exploration and encoding is performed at the expense of the motor task in older adults. On the other hand, the speed reduction could be interpreted as a strategic precaution measure of older adults. Given the need to share resources, these participants could slow down to maintain stability. Whether this is an objectively effective strategy or not, for older adults a speed reduction implies a more adequate tactic to adapt to potential postural threats or perturbations (Menz et al., 2003). Our interpretation of CMI in this study is based on the ecological quality of the tasks being accomplished, i.e., the tight relationship between visual and postural behavior. We consider older adults’ walking speed reduction as a postural adaptation in order to accomplish the task of learning the unknown environment, analogous to observations of postural stabilization at the service of accomplishing supra-postural visual tasks (Stoffregen et al., 2000). It may also be that gait modulation occurred not only due to age-related increase in cognitive resource-sharing but also to compensate for age-related deficits in cognitive processing speed (Salthouse, 2000). Reductions in processing speed has also been linked to reductions in walking speed with age (Soumaré et al., 2009). While we did not find a significant correlation between the change in walking speed and processing speed, as evaluated via the first part of the Trail Making Test (*r* = 0.40, *p* = 0.047 > *p_*corrected*_* = 0.008), the general trend was positive, indicating a greater walking speed reduction for individuals who took longer to complete the test. Moreover, older adults performed worse on this screening test compared to young adults (see Table 2).

Decades of research revealed that both spatial learning (Lindberg and Gärling, 1982; Albert et al., 1999; Rand et al., 2015) and postural control (Woollacott and Shumway-Cook, 2002; Yogev-Seligmann et al., 2008) are attentionally demanding. Beyond older adults’ mobility issues (due to both central and peripheral age-related alterations, Seidler et al., 2010; Paraskevoudi et al., 2018), which call for greater cognitive resources, it is also well established that older individuals have more difficulty on spatial learning and memory tasks, especially regarding new environments (Iachini et al., 2009; Moffat, 2009). One must intentionally attend to the environment in order to encode spatial relations, and while landmark and boundary learning may require little attention, associating such information to actions or locations within the environment is more demanding (Chrastil and Warren, 2012). In addition, spatial updating, which contributes to spatial learning, relies on the congruence of allothetic and idiothetic information. While walking through an environment, this updating of a set of self and object relations may be further affected by aging, given age-related issues in the incremental learning and the accuracy of older adults’ internal body representations (Personnier et al., 2010; Boisgontier and Nougier, 2013; Lafargue et al., 2013).

If in our setting visual exploration and encoding were competing for cognitive resources with postural control while walking, we should observe a change in navigation and learning performance as a function of walking speed over trials. To explore this, we focused on the trials where learning took place, excluding the first one and setting a criterion based on trajectory efficiency (see section “Materials and Methods”). We found that the adaptation indices calculated for the trajectory efficiency and proportion of goal fixations were positively correlated with the adaptation index of walking speed. In other words, the modulation of our measures of spatial learning (trajectory efficiency and goal fixations) covaried with the modulation of walking speed over the trials where learning occurred. The proportion of goal fixations with respect to total fixations is an indication of visual sampling and, to a certain extent, visual search efficiency. In our context, the modulation in fixation distributions from one trial to the next while learning can be considered as an indication of familiarity with the environment. Trajectory efficiency, on the other hand, is a more direct way of establishing whether participants have learnt the location of the goal. These positive correlations demonstrate that as participants learn the location of the goal with respect to visual elements of the environment, there is a common evolution between their walking speed, scanning behavior and navigation trajectory. Importantly, these findings are based on trials where learning took place, excluding the first one, thus avoiding a potential bias due to more extreme behavior observed upon the first encounter with the novel environment – in particular for older adults.

The cognitive demands of motor tasks interacting with spatial learning have been observed in previous studies (Kerr et al., 1985; Lövdén et al., 2005; Taillade et al., 2013; Rand et al., 2015; Barhorst-Cates et al., 2017). Though methods differed, all these former studies revealed an improvement in spatial memory or wayfinding when motor task demands were reduced. Our findings therefore agree with the consensus on the increased CMI in older age, extending findings to the field of navigation and supporting the limited literature therein. Importantly, this agreement is within an ecological environment, closer to real-world settings, thus making these findings novel with respect to the literature on both aging spatial navigation and CMI, which tend to lack ecological validity. Ultimately, daily activities have a multisensory and complex cognitive nature, therefore the CMI we interpret is indicative of real-word actions and in line with the observation that natural walking is actually analogous to laboratory-tested dual task walking (Hillel et al., 2019). Our study calls for future, more explicit (and ecologically valid) research to consider the cognitive demands of postural control in aging spatial navigation, and this would further elaborate current theories of cognitive aging as well (e.g., Kuehn et al., 2018). Moreover, our approach invokes another question, which, to our knowledge, has yet to be addressed: where is the line drawn in terms of sufficient information for safe and successful navigation in older age? We know that cognitive and sensorimotor functions degrade with age and that sensory integration is both more important but also more difficult for older adults (de Dieuleveult et al., 2017). And while ours and the aforementioned spatial learning studies highlight the cognitive burden of postural control for older adults, idiothetic – and especially podokinetic (Chrastil and Warren, 2013) – cues are crucial for successful navigation and spatial learning (Mittelstaedt and Mittelstaedt, 2001; Ruddle and Lessels, 2006; Ruddle et al., 2011; Chrastil and Warren, 2012). To explore this, it would be interesting to assess the effect of modulating the level of resource requirements in each of the cognitive spatial and postural domains via a grading of task difficulty. Furthermore, the study of cognitive-motor interactions during ecological navigation tasks can lead to the development of appropriate navigation aids which are not attentionally taxing (Klatzky et al., 2006; Schellenbach et al., 2010; Gardony et al., 2013, 2015) for older adults, but also for individuals prone to cognitive resource-sharing issues more generally.

### Neuroanatomical Correlates of the Change in Walking Speed

Given the neuroimaging evidence of greater cognitive involvement in walking with older age (Zwergal et al., 2012; Hamacher et al., 2015) and the differential recruitment of brain areas under CMI (Leone et al., 2017), we explored possible neuroanatomical correlates of our hypothesized CMI during spatial learning. More specifically, the evidence of regional atrophy being linked to slowing of gait and the fact that gray matter volume accounts for functional changes in cerebral activity in older age (Kalpouzos et al., 2012) led us to examine whether we could find associations between age-related changes in brain structures and walking speed in an ecological context. The regression analysis on grey matter (GM) volume in a subset of our population revealed interesting and pertinent results. The change in walking speed between the first and last trials was negatively correlated with GM volume in parts of the superior parietal lobule, superior aspect of the occipital lobe on the lateral surface and precuneus. These loci are neighboring association areas lying near the border of the occipital and parietal lobes and they are indeed part of the network involved in locomotor control (Wang et al., 2008), including visually-guided walking (Malouin et al., 2003) and they are associated with walking speed (Callisaya et al., 2014; Blumen et al., 2019). Importantly, these areas are involved in processes that are crucial for spatial navigation. Posterior parietal areas, including the superior parietal lobule and precuneus, play an essential role in visuo-spatial imagery and attention, episodic memory, self-processing and internal representations of the self and the environment (Corbetta et al., 1995; Wolpert et al., 1998; Suchan et al., 2002; Cavanna and Trimble, 2006). Further, recruitment of the precuneus was shown during explicit learning in young, but not older adults (Dennis and Cabeza, 2011). Along with the superior aspect of the occipital lobe (collectively referred to as the superior parieto-occipital cortex), these areas also participate in the encoding or retrieval/imagery of space during navigation tasks, in both egocentric and allocentric reference frames, thus contributing to spatial learning (Committeri et al., 2004; Frings et al., 2006; Gramann et al., 2010; Weniger et al., 2010; Barra et al., 2012).

There is also support for the relevance of our findings with respect to the literature on the neural correlates of CMI while walking. In two studies examining GM volume associated with dual task walking in older adults, the superior aspect of the occipital region was found to be part of a network linked to walking speed (Allali et al., 2019), while all three of our identified areas were involved in a network associated with the dual task cost (the change between single and dual task performance) in walking speed (Tripathi et al., 2019). Furthermore, functional neuroimaging studies found increased activation of the precuneus (Blumen et al., 2014) and superior parietal area (Bürki et al., 2017) for dual task walking in older adults. Given the common finding of prefrontal involvement in dual task walking in older adults (Holtzer et al., 2011; Blumen et al., 2014; Wagshul et al., 2019), it could appear surprising that such an association was not found in our study. However, methodological differences in neuroimaging studies (whether it is the imaging technique, walking condition or the nature of the cognitive task) limit direct comparisons. Furthermore, it is reasonable to assume that there is no brain region responsible *specifically* for cognitive-motor interference, but that this would rather depend on the demands of each task, the overlap in neural pathways recruited as well as the individual’s expertise, cognitive and motor abilities (Leone et al., 2017), similar to the variability found in dual task behavioral findings (Yogev-Seligmann et al., 2012).

It is noteworthy that the correlations between our variables of walking speed modulation (the change in walking speed and adaptation index of walking speed) and neuropsychological tests screening for visuo-cognitive abilities did not reach significance, yet a relationship was found with GM atrophy. We see therefore that slowing of gait in a task as ecological and natural as is visually exploring a novel environment may be linked to subclinical structural brain alterations, while the association of dual task cost or walking speed reduction with cognitive functions that is often reported in the literature (Montero-Odasso et al., 2012; Killane et al., 2014) is not yet manifested. Furthermore, the atrophy associated with the change in walking speed in our study was within parieto-occipital regions that are actually linked to gait variability in older adults with higher fall risk (Fernandez et al., 2019), early-onset Alzheimer’s disease (Karas et al., 2007), and mild cognitive impairment (Doi et al., 2017). Thus, albeit exploratory given the relatively small size of our population, these findings are coherent with the task and they are also in line with the literature on spatial cognition and aging in the control of walking. Further studies are warranted in order to better characterize the neural bases of age-effects on spatial learning and navigation, taking also into account the differential contribution of brain areas to postural control with age. For example, in addition to the parietal regions we identified, the degeneration in areas that are key for spatial navigation and learning, such as the hippocampus (Driscoll et al., 2003), is also associated with age-related gait alterations, notably slower walking speed (Ezzati et al., 2015).

### Limits, Strengths and Perspectives

The sample size of our study is a limitation we must acknowledge. To account for the limited population sample used for our volumetric study, we adopted very conservative statistical thresholds and we chose a whole-brain exploratory analysis. Despite these restrictions, we found strong correlations with areas that are tightly linked to the two tasks that we hypothesized would be interfering with one another. These results should nevertheless be interpreted with some caution as they would not reach significance had we used an even more restrictive threshold [a voxel-wise threshold of *p* < 0.01 family-wise error corrected, as suggested by (Eklund et al., 2016)]. Further research is therefore warranted, with a larger sample size. We should also acknowledge that the navigation study was not designed to specifically test our hypothesis, and therefore other interpretations might be considered. One could perhaps argue that older adults’ slowing of gait on the first trial was a result of apprehension or stress in the context of a scientific experiment, or simply due to discomfort with the worn equipment. It is possible that older adults were more mindful of wanting to perform well and therefore slowed down at the start of the experiment, while anxiety can indeed influence attentional resources during locomotion (Gage et al., 2003). Nevertheless, the correlations between the adaptation indices and the neural correlates, which are specific to spatial orientation and sensory integration, do lend support to our hypothesis. Furthermore, our older adults did not have a more anxious profile (no significant difference with young adults on the state-trait anxiety inventory) and there was no significant correlation between the change in walking speed and participants’ score on the state-trait anxiety inventory.

Finally, the ecological and epidemiological relevance of our findings should be noted. Visually exploring an environment while walking is a task inherent to daily behavior. As such, at least to our knowledge, it has not been considered so taxing as to affect postural control, as our results could let interpret. Although some studies examined the effect of cognitive load on visuo-motor behavior (Ellmers et al., 2016), what we propose is rather to rethink about cognitive resource-sharing in such tasks. The act of visual exploration alone (to memorize an environment or avoid obstacles for example) may interfere with postural control, without increasing cognitive load via an additional task, which is what we found within a sample of healthy, active, community dwelling older adults. Indeed, visual scanning to encode an environment requires higher order cognitive processes, such as working memory, visual attention, and monitoring and updating one’s internal representation of the environment, the relationship between visual elements but also between these and one’s self. Future studies on navigation in aging are needed to take into account cognitive-motor interactions, to better understand how cognitive and sensorimotor aging influences older adults’ abilities. A better understanding of CMI in the context of spatial navigation and reorientation would help improve the development of navigation and mobility aids, as well as training protocols that help older and frailer adults regain or maintain their autonomy. Moreover, the fact that this study was performed in a real, street-like environment where the task resembles real-life navigation challenges (trying to encode and memorize an unknown location) adds ecological validity that is mostly lacking in both navigation (Schöberl et al., 2020) and dual task CMI (see Table 1 in Smith et al., 2016) protocols.

### Conclusion

While the dual task paradigm is the prevalent means of investigating cognitive-motor interference while walking in older adults, our study questions what may constitute a dual task in the context of a common activity. Importantly, we wish to highlight the cognitive load of postural control in the context of aging research on navigation, as this aspect of navigation ability is rarely considered. We proposed that visually and physically exploring an unknown, real environment, with the aim of learning its layout, may be subject to cognitive-motor interference for older adults. Although our findings may not be conclusive for the interpretation of cognitive-motor interference, the indicators we have observed invite future research on the matter, particularly given that older adults are uncomfortable in unfamiliar environments (Rosenbaum et al., 2012; Phillips et al., 2013). This apprehension to novel environments can lead to more sedentary behavior, which in turn has a negative impact on both cognitive and mobility functions. To conclude, several encouraging intervention studies have revealed improvement in cognitive and mobility functions, and even in terms of brain volumetric and activity increases (Li et al., 2018). Our findings may therefore be used to support the elaboration of personalized training, assessment and assistance. If cognitive-motor interference and associated structural brain changes can be detected during a seemingly “simple” task in a healthy, active population as are the members of our cohort, future research may consider simpler diagnostic tools and the development of navigation aids for autonomy maintenance in older adults that are appropriate for their daily needs.

## Data Availability Statement

The data analyzed in this study is subject to the following licenses/restrictions: The original data analyzed in this article were acquired for a previous study of ours (Bécu et al., 2020; Nat Hum Behav). The data are available upon request. Requests to access these datasets should be directed to AA: angelo.arleo@inserm.fr and CA: catherine.agathos@sorbonne-universite.fr.

## Ethics Statement

The studies involving human participants were reviewed and approved by CPP Ile de France V (ID_RCB 2015-A01094-45, n. CPP: 16122 MSB). The patients/participants provided their written informed consent to participate in this study. Written informed consent was obtained from the individual(s) for the publication of any potentially identifiable images or data included in this article.

## Author Contributions

CA, MB, DB, and AA conceived the study. SR, MB, and CH collected the data. CA, SR, and MB analyzed the data. CA, SR, MB, DB, and AA wrote the manuscript. All authors contributed to the article and approved the submitted version.

## Conflict of Interest

DB is employed by Essilor Canada Ltd as a senior vision scientist and research project manager. The remaining authors declare that the research was conducted in the absence of any commercial or financial relationships that could be construed as a potential conflict of interest.

## Footnotes

1 http://www.streetlab-vision.com

2 www.fil.ion.ucl.ac.uk/spm/

3 http://www.neuro.uni-jena.de
