# Supplementary material for: Postural Control While Walking Interferes With Spatial Learning in Older Adults Navigating in a Real Environment
Source: Front Aging Neurosci. 2020 Nov 12;12:588653. doi: 10.3389/fnagi.2020.588653 (PMC7689348; doi:10.3389/fnagi.2020.588653)
Supplement: Supplementary file 1 [file Data_Sheet_1.docx]

**Supplementary Material**

**Statistical Power Analysis for the Voxel-Based Morphometry Study**

To support the voxel-based morphometry (VBM) analysis, we performed a power calculation based on an independent data set involving thirty participants (15 young and 15 healthy older adults, age range: 30 to 84 years). First, we performed a Power analysis using the [G*Power3 Sofware](http://www.gpower.hhu.de) (Faul et al., 2007). In this analysis, the 1-beta value is computed as a function of the sample sizes and the population effect size parameter (Figure 1). We plotted the conventional values for the effect size *d* (small *d* = 0.2, medium *d* = 0.5, large *d* = 0.8). The result showed that our sample size of 30 participants was theoretically large enough for the detection of a large effect size with enough statistical power (i.e. 80%) but not for the detection medium and small effect sizes.

*
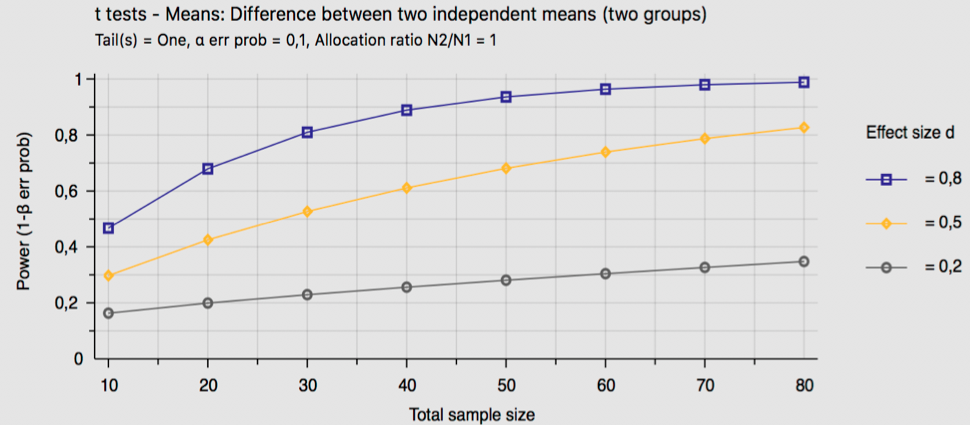
*

Figure 1. Post-hoc analysis performed on G*Power3

However, the calculation methods used on G*Power3 are too conservative and not appropriate in the case of MRI studies, as emphasized by C. Gaser, the designer of the VBM toolbox on the [SPM-list](https://www.jiscmail.ac.uk/cgi-bin/webadmin?A2=spm;8f3fc98b.0901) and in several articles (Hayasaka et al., 2007; Mumford and Nichols, 2008; Joyce and Hayasaka, 2012). Indeed, determining the power of an fMRI or morphological study is a formidable task. An array of parameters must be specified, such as the within and between-subject variance, the first and second level design, the temporal autocorrelation and the size of the hypothesized effect, all of which may vary voxel by voxel (Durnez et al., 2016). To deal with this complexity we performed a second power analysis using the [Powermap software](https://sourceforge.net/projects/powermap/) (Joyce and Hayasaka, 2012) specially designed for neuroimaging studies. Here, power is calculated “around” each voxel rather than “at” each voxel, meaning that the calculated power reflects the probability of detecting activation in a neighborhood centered at each voxel (due to spatial correlation among voxels). We obtained sample size images allowing us to visualize the ability to detect differences voxel by voxel between our groups with respect to sample size, here for a power (1-beta) fixed at 80% (Figure 2).

The results suggested that our sample size of 18 participants was sufficient to detect an important portion of differences in GM (indicated in blue in Figure 2). This pattern is consistent with the three significant clusters showing large effect sizes provided by our multiple regression analysis with *r* correlation values ranging from -0.68 to -0.90 (*r* values greater than 0.5 are considered to indicate large effect sizes). Nevertheless, the above analysis also showed that our sample size did not allow us to detect subtle changes related to brain aging, for example in a few frontal regions (indicated in red in Figure 2). To detect these subtle changes, assuming a statistical power at 80%, we would need to add several hundred participants as indicated in Figure 1 as well.

*
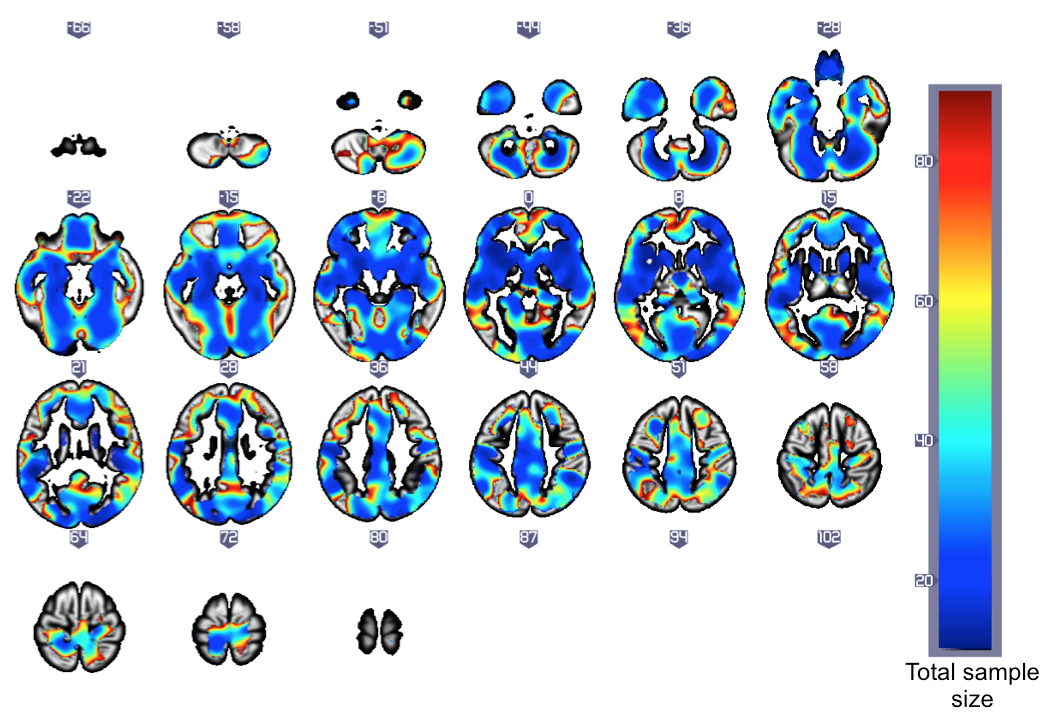
*

Figure 2. Power analysis performed on PowerMap showing detection power of differences as function to the sample size for a power (1-beta) fixed at 80%

The sample size used in our study seems to be sufficient to detect an important part of brain differences in terms of GM changes with a power of the order of 80% or 90% if the probabilities of difference between the 2 groups are sufficiently separated (between 15% and 20% of difference (Bhaumik et al., 2009)). In addition, our sample size is similar to that of other recent morphological studies such as (Simon et al., 2013; Sato et al., 2015). Nevertheless, our number of participants appears to be clearly insufficient to detect more subtle changes. In addition, our study is not powerful enough to detect a putative differential pattern of brain structure changes between genders (Sampedro et al., 2015) such as recently reported by the ADNI group (Weiner et al., 2017).

**References**

Bhaumik, D. K., Roy, A., Lazar, N. A., Kapur, K., Aryal, S., Sweeney, J. A., et al. (2009). Hypothesis testing, power and sample size determination for between group comparisons in fMRI experiments. *Stat. Methodol.* 6, 133–146. doi:10.1016/j.stamet.2008.05.003.

Durnez, J., Degryse, J., Moerkerke, B., Seurinck, R., Sochat, V., Poldrack, R. A., et al. (2016). Power and sample size calculations for fMRI studies based on the prevalence of active peaks. *bioRxiv*, 49429. doi:10.1101/049429.

Faul, F., Erdfelder, E., Lang, A.-G., and Buchner, A. (2007). G* Power 3: A flexible statistical power analysis program for the social, behavioral, and biomedical sciences. *Behav. Res. Methods* 39, 175–191. doi:10.3758/BF03193146.

Hayasaka, S., Peiffer, A. M., Hugenschmidt, C. E., and Laurienti, P. J. (2007). Power and sample size calculation for neuroimaging studies by non-central random field theory. *Neuroimage* 37, 721–730. doi:10.1016/j.neuroimage.2007.06.009.

Joyce, K. E., and Hayasaka, S. (2012). Development of PowerMap: a software package for statistical power calculation in neuroimaging studies. *Neuroinformatics* 10, 351–365. doi:10.1007/s12021-012-9152-3.

Mumford, J. A., and Nichols, T. E. (2008). Power calculation for group fMRI studies accounting for arbitrary design and temporal autocorrelation. *Neuroimage* 39, 261–268. doi:10.1016/j.neuroimage.2007.07.061.

Sampedro, F., Vilaplana, E., De Leon, M. J., Alcolea, D., Pegueroles, J., Montal, V., et al. (2015). APOE-by-sex interactions on brain structure and metabolism in healthy elderly controls. *Oncotarget* 6, 26663. doi:10.18632/oncotarget.5185.

Sato, K., Kirino, E., and Tanaka, S. (2015). A voxel-based morphometry study of the brain of university students majoring in music and nonmusic disciplines. *Behav. Neurol.* 2015. doi:10.1155/2015/274919.

Simon, A. B., Griffeth, V. E. M., Wong, E. C., and Buxton, R. B. (2013). A Novel Method of Combining Blood Oxygenation and Blood Flow Sensitive Magnetic Resonance Imaging Techniques to Measure the Cerebral Blood Flow and Oxygen Metabolism Responses to an Unknown Neural Stimulus. *PLoS One* 8, e54816. doi:10.1371/journal.pone.0054816.

Weiner, M. W., Veitch, D. P., Aisen, P. S., Beckett, L. A., Cairns, N. J., Green, R. C., et al. (2017). The Alzheimer’s Disease Neuroimaging Initiative 3: Continued innovation for clinical trial improvement. *Alzheimer’s Dement.* 13, 561–571. doi:10.1016/j.jalz.2016.10.006.
